# Mechanically resolved imaging of bacteria using expansion microscopy

**DOI:** 10.1371/journal.pbio.3000268

**Published:** 2019-10-17

**Authors:** Youngbin Lim, Anthony L. Shiver, Margarita Khariton, Keara M. Lane, Katharine M. Ng, Samuel R. Bray, Jian Qin, Kerwyn Casey Huang, Bo Wang

**Affiliations:** 1 Department of Bioengineering, Stanford University, Stanford, California, United States of America; 2 Department of Chemical Engineering, Stanford University, Stanford, California, United States of America; 3 Department of Microbiology and Immunology, Stanford University School of Medicine, Stanford, California, United States of America; 4 Chan Zuckerberg Biohub, San Francisco, California, United States of America; 5 Department of Developmental Biology, Stanford University School of Medicine, Stanford, California, United States of America; The University of Oregon, UNITED STATES

## Abstract

Imaging dense and diverse microbial communities has broad applications in basic microbiology and medicine, but remains a grand challenge due to the fact that many species adopt similar morphologies. While prior studies have relied on techniques involving spectral labeling, we have developed an expansion microscopy method (μExM) in which bacterial cells are physically expanded prior to imaging. We find that expansion patterns depend on the structural and mechanical properties of the cell wall, which vary across species and conditions. We use this phenomenon as a quantitative and sensitive phenotypic imaging contrast orthogonal to spectral separation to resolve bacterial cells of different species or in distinct physiological states. Focusing on host–microbe interactions that are difficult to quantify through fluorescence alone, we demonstrate the ability of μExM to distinguish species through an in vitro defined community of human gut commensals and in vivo imaging of a model gut microbiota, and to sensitively detect cell-envelope damage caused by antibiotics or previously unrecognized cell-to-cell phenotypic heterogeneity among pathogenic bacteria as they infect macrophages.

## Abstract 2

This study describes a new expansion microscopy imaging technique that uses cell wall mechanics as a quantitative and phenotypic imaging contrast, orthogonal to spectral separation, in order to resolve bacterial cells of different species or in distinct physiological states.

## Introduction

Imaging of heterogeneous bacterial populations has broad applications in understanding the complex microbiota that exist on and within our bodies, as well as complex host–microbial interfaces, yet remains a significant challenge due to the lack of suitable tools for distinguishing species and identifying altered physiological states [1–3]. Analyses to date have mostly relied on spectral separation using fluorescence in situ hybridization (FISH) with probes designed to target 16S RNA sequences specific to certain taxa [4], or genetically engineered microbes that express distinct fluorescent proteins [5]. However, these methods are generally insensitive to physiological changes in bacterial cells that are often modulated by host environments and believed to be critical for the growth and spatial organizations of microbes [6,7].

The bacterial cell wall is a macromolecule responsible for shape determination in virtually all bacteria. Although little is known about the molecular architecture of the cell wall in most non-model organisms, its dimensions can vary widely, with the wall typically thick (tens of nanometers [8,9]) in gram-positive species and thin (approximately 2 to 4 nm [10]) in gram-negative species, and wall rigidity can vary across Young’s moduli of between 10 and 100 MPa [11,12]. The cell wall also has various biochemical compositions [13] and exhibits distinct spatial patterns of cross-linking density [14], molecular organization [15,16], thickness [8,9], and stiffness [11,12], all of which depend on species and cell physiology. Thus, cell wall mechanics can potentially provide a contrast that is orthogonal to spectral separation in distinguishing species and even cellular physiological states. However, while cell wall structure and mechanics have been measured by electron microscopy and atomic force microscopy [8,10,17], these methods are low-throughput and incompatible with dense bacterial populations or in vivo applications.

To address this challenge, we develop a new method that extends the application of expansion microscopy (ExM) to bacteria, particularly in the contexts of multispecies communities and infection. ExM is a recently developed optical imaging method that was designed to provide superior resolution to traditional fluorescence imaging, but its applications to bacteria have so far been limited [18,19]. ExM relies on simple chemistry, in which biomolecules are anchored to a swellable polyelectrolyte hydrogel network, and then cell membranes are permeabilized by detergents while proteins are digested by proteases to produce homogenous mechanical properties across the sample. The hydrogel networks are then expanded in pure water through osmotic forces and electrostatic repulsion between polyelectrolyte chains. The expanded sample can be imaged using conventional optical microscopy and digitally compressed to gain resolution. While prior studies have focused on achieving uniform expansion of samples, primarily in mammalian cell and animal tissues [19–21], we posited that differences in the expandability of the bacterial cell wall, which would not be digested through typical ExM protocols, could provide imaging contrast that reflects its molecular structure and distinct mechanical properties.

In this study, we show that the peptidoglycan cell wall requires additional treatments to be broken down for expansion. We demonstrate, with both in vitro and in vivo applications, that the contrast in cell wall expandability is sufficient to resolve different bacterial species within complex and dense communities. In addition, this method is sensitive enough to detect cell wall damages caused by antibiotics or host defensive responses that are otherwise difficult to capture using traditional imaging methods. We anticipate that this method will enable future research in three major areas: (1) super-resolution imaging of bacterial subcellular components [22] without the need for expensive optics or special fluorophores, (2) high-content imaging that integrates both spectral and mechanical contrasts to dissect complex microbial communities, and (3) in vivo phenotyping of cell wall mechanics and integrity [23].

## Results

### Expansion provides quantitative imaging contrast to distinguish bacterial species

**Fig 1:**
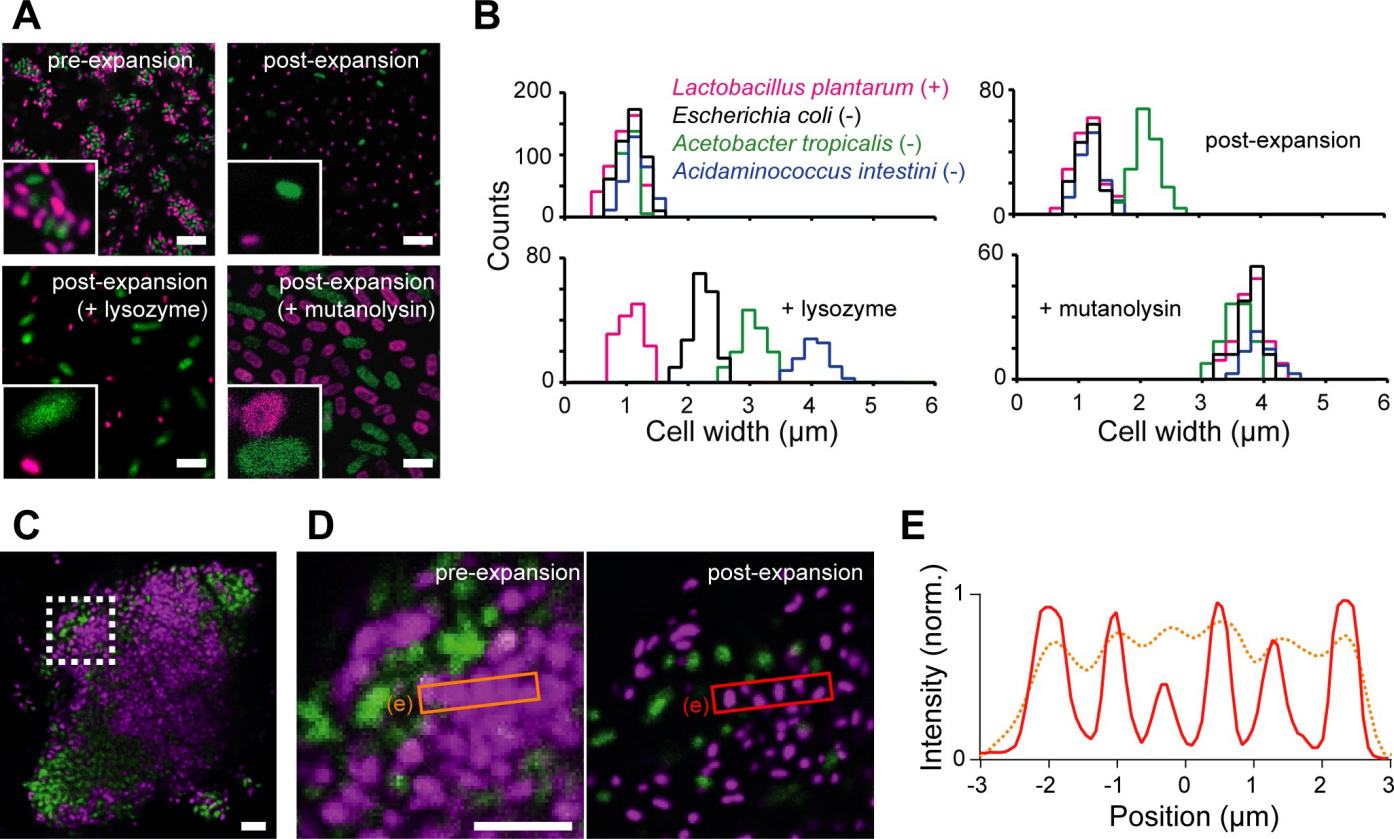
Differential expansion provides novel imaging contrast in μExM. (A) Representative confocal fluorescence images of a mixture of mCherry–*L*. *plantarum* and GFP–*A*. *tropicalis*. Top: untreated cells pre- and post-expansion using the original ExM protocol; bottom left: cells treated with lysozyme to partially digest the bacterial cell wall before expansion; bottom right: cells treated with mutanolysin to fully digest the cell wall before expansion. Dark area at the center of the cells is occupied by condensed chromosome [26,27]. Insets: magnified views to show cell size differences. (B) Quantification of cell width distributions before and after expansion for representative microbial species. Lysozyme treatment maximizes the contrast in expansion between species, while mutanolysin treatment expands all species approximately 4-fold. When fluorescently labeled strains were not available, we measured the expansion ratio using DNA staining. Plus sign (+) and minus sign (−) denote gram-positive and gram-negative, respectively. All histograms were generated using data collected from at least five maximum intensity projection images from at least two independent experiments. The data underlying this figure are included in S1 Data. (C) Pre-expansion image of a microcolony of mCherry–*L*. *plantarum* and GFP–*A*. *tropicalis*. (D) Magnified view of the region highlighted by the dashed box in (C) before expansion (left) and after mutanolysin treatment and expansion (right). The scale bar in the post-expansion image has been rescaled to match the pre-expansion dimensions. (E) Cross-sectional normalized fluorescence intensity profiles of the regions highlighted by the boxes in (D), showing that μExM preserves the relative positions of cells (peaks in the orange and red curves overlap). The data underlying this figure are included in S2 Data. All images are maximum intensity projections. Scale bars, 10 μm in (A), 5 μm in (C) and (D). ExM, expansion microscopy; GFP, green fluorescent protein; μExM, expansion microscopy of microbes; norm., normalized.

To test whether the cell wall indeed restricts expansion in ExM, we imaged a mixture of two common symbiotic bacteria isolated from the gut of the fruit fly *Drosophila melanogaster*, *Acetobacter tropicalis* (green fluorescent protein [GFP] labeled), and *Lactobacillus plantarum* (mCherry labeled) [24] using a standard ExM protocol (**Fig 1A**, top) [25]. We quantified the expansion ratio of individual cells based on cell width, because width can be measured precisely regardless of cellular orientation in three dimensions. We found that *A*. *tropicalis* and *L*. *plantarum* cells only expanded approximately 1.9- and 1.2-fold, respectively (**Fig 1B**, top), both of which were smaller than the 4-fold expansion expected from previous characterizations of ExM [19,25].

We then digested the wall using lysozyme from chicken egg white or mutanolysin from *Streptomyces globisporus*, after permeabilizing the cell membrane with methanol. Both enzymes are muramidases that cleave 1,4-beta-linkages between N-acetylmuramic acid and N-acetyl-D-glucosamine residues in the cell wall but differ in mechanism of action and activity [28]. Overnight lysozyme treatment enhanced the contrast in expansion ratios between species (**Fig 1A and 1B**, bottom left): *A*. *tropicalis* cells were fully expanded approximately 4-fold, whereas *L*. *plantarum* cells were largely unaffected. By contrast, mutanolysin treatment led to 4-fold expansion of both species (**Fig 1A and 1B**, bottom right). The uniform expansion enabled us to resolve individual cells in densely packed mixed colonies of the two species while preserving their relative positions (**Fig 1C–1E**).

Next, we tested whether these findings could be extended to other commensal species from the human gut. We applied the method to nine species, including *Acidaminococcus intestini*, *Bacteroides finegoldii*, *Bacteroides ovatus*, *Bifidobacterium breve*, *Citrobacter* sp., *Clostridium innocuum*, *Escherichia coli*, *Parabacteroides distasonis*, and *Salmonella enterica*. These species were chosen to include gram-positives and gram-negatives. For all species, the expansion ratios were approximately 4-fold after mutanolysin treatment, whereas the ratios after lysozyme treatment varied widely (**Fig 1B**, bottom left), particularly among gram-negatives. They were generally larger for all gram-negative species than for gram-positives (**Table 1** and **S1 Fig**). Together, these results indicate that the extent of breakdown of the cell wall determines the expansion of bacterial cells, and the expansion ratio provides quantitative and fine resolution in distinguishing species beyond the traditional classification of gram-negatives and gram-positives. Henceforth, we refer to this method involving lysozyme or mutanolysin digestion as μExM, expansion microscopy of microbes.

**Table 1:** Expansion of bacteria is species specific.

Strains	Culture medium	Culture condition	Antibiotic resistance	Expansion ratio^4^
*Lactobacillus plantarum*^1^	MRS	Aerobic, 30°C	chloramphenicol	1.12 ± 0.03
*Acetobacter tropicalis*^2^	MRS	Aerobic, 30°C	tetracycline	3.09 ± 0.07
*Escherichia coli*^2^ (DH5-alpha)	LB	Aerobic, 37°C	ampicillin	2.21 ± 0.04
*E*. *coli imp4213*^2^	LB	Aerobic, 37°C	kanamycin	N.D.
*Salmonella enterica*^2^ (SL12023)	LB	Aerobic, 37°C	ampicillin	2.23 ± 0.05
*Bifidobacterium breve*^1^^,^^3^	RCM	Anaerobic, 37°C	N.A.	1.21 ± 0.04
*Clostridium innocuum*^1^^,^^3^	RCM	Anaerobic, 37°C	N.A.	1.14 ± 0.03
*Bacteroides ovatus*^2^	GAM	Anaerobic, 37°C	N. A.	2.56 ± 0.02
*Bacteroides finegoldii*^2^^,^^3^	GAM	Anaerobic, 37°C	N.A.	3.67 ± 0.07
*Acidaminococcus intestini*^2^^,^^3^	GAM	Anaerobic, 37°C	N.A.	3.98 ± 0.09
*Parabacteroides distasonis*^2^^,^^3^	GAM	Anaerobic, 37°C	N.A.	3.79 ± 0.09
*Citrobacter* sp.^2^	YCFA	Anaerobic, 37°C	N.A.	3.89 ± 0.02

^1^Gram-positive (gray shaded).

^2^Gram-negative.

^3^Obtained through BEI Resources, NIAID, NIH, as part of the Human Microbiome Project: *B*. *breve* strain HPH0326, HM-856; *C*. *innocuum* strain 6_1_30, HM-173; *B*. *ovatus* strain 3_8_47FAA, HM-222; *B*. *finegoldii* strain CL09T03C10, HM-727; *A*. *intestini* strain D21, HM-81; *P*. *distasonis* strain 31_2, HM-169 (previously deposited as *Porphyromonas* sp.); *Citrobacter* sp. strain 30_2, HM-34. For coculture experiments, *B*. *breve* strain JCP7499, HM-1120 was used instead of HPH0326.

^4^The expansion ratio was computed as the average cell width in post-expansion images of lysozyme-treated cells divided by the average cell width in pre-expansion images. The expansion ratios are reported as mean ± SEM determined from ≥5 confocal images, each containing hundreds of cells, collected from at least two independent experiments.

Abbreviations: GAM, Gifu Anaerobic Medium; LB, Lysogeny Broth; MRS, De Man, Rogosa, and Sharpe; N. A., not available; N.D., not determined; RCM, Reinforced Clostridial Medium; SEM, standard error of the mean; YCFA, Yeast extract-Casitone-Fatty Acid.

**Fig 2:**
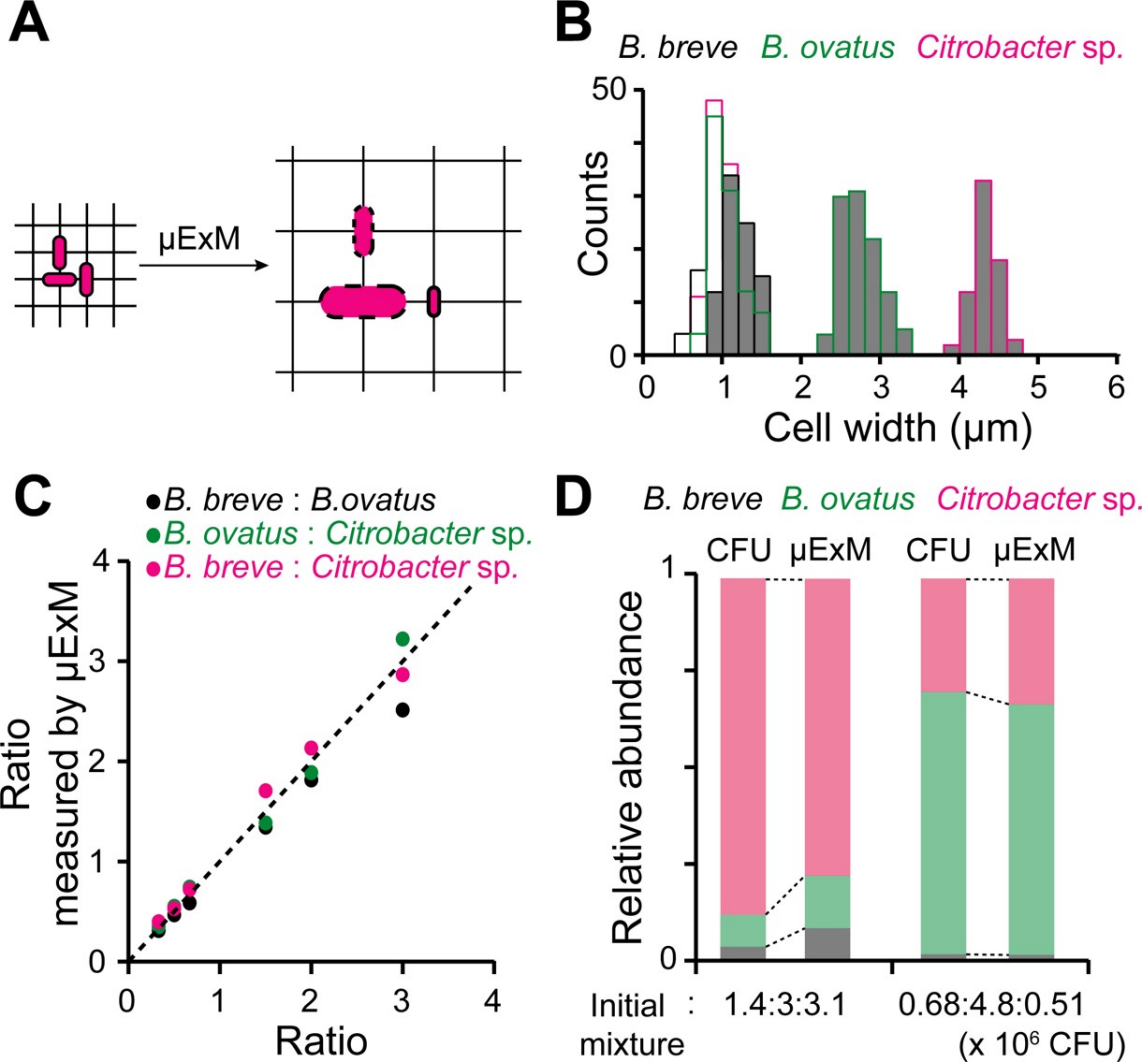
μExM accurately quantifies species composition in an in vitro defined community of human gut commensals. (A) Schematic showing that differential expansion provides imaging contrast to distinguish species in mixed populations that cannot be labeled with species-specific fluorescent tags. (B) Distribution of cell widths for human commensal species, *B*. *breve*, *B*. *ovatus*, and *Citrobacter* sp., before (open bars) and after expansion upon lysozyme digestion (gray bars). Cell widths were measured by visualizing the DNA stain TO-PRO-3. Note that cell widths fully overlap before expansion but become well separated after expansion. The data underlying this figure are included in S3 Data. (C) Comparison of cell-count ratios for the three species based on classification using μExM width measurements with the mixing ratios of each pair. Each symbol color represents a pairwise comparison. Cultures of individual species were fixed separately, mixed at cell number ratios of 1:2:3, 3:1:2, and 2:3:1 (*B*. *breve*: *B*. *ovatus*: *Citrobacter* sp.), and then imaged through μExM. The data underlying this figure are included in S4 Data. (D) Quantification of relative species abundance in the three-member community after 2.5 h of coculturing, starting from two initial mixtures, as measured by μExM and CFU counts. The initial mixture compositions are reported in CFU counts (*B*. *breve*: *B*. *ovatus*: *Citrobacter* sp.). The data underlying this figure are included in S5 Data. CFU, colony-forming unit; μExM, expansion microscopy of microbes.

Our findings suggest that μExM both improves imaging resolution, as shown in previous studies [19–21,25], and provides an additional imaging contrast associated with cell wall mechanical properties that is orthogonal to spectral separation commonly used in fluorescence microscopy. Moreover, this contrast can be amplified by partial wall digestion using lysozyme. We posited that in some cases, the contrast in expansion should be sufficient to distinguish between microbial species with differing cell wall properties (**Fig 2A**). For example, the post-lysozyme expansion ratio distributions of four species, *L*. *plantarum*, *E*. *coli*, *A*. *tropicalis*, and *A*. *intestini*, were clearly distinct, despite the variation across cells of the same genotype (**Fig 1B**, bottom left). To further test our hypothesis, we chose three human commensal species (*B*. *breve*, *B*. *ovatus*, and *Citrobacter* sp.), as they are of similar pre-expansion size and morphology and thus difficult to distinguish by traditional methods. Importantly, all these strains are currently not amenable to genetic transformation, and therefore species-specific fluorescent-protein labeling has not been possible. Following lysozyme treatment and expansion, the three species can be easily separated based on post-expansion size using DNA staining (**Table 1** and **Fig 2B**). This size-dependent expansion allowed us to quantify precisely their abundance in both mixed populations (**Fig 2C**) and cocultured communities (**Fig 2D**), as validated through colony-forming unit (CFU) measurements. These results demonstrate the utility of μExM for studying the assembly and growth of mixed bacterial populations.

### Expansion detects cell wall damage induced by antibiotics with high sensitivity

Given that expansion is dependent upon cell wall properties, we reasoned that μExM should also reveal different expansion phenotypes in cells grown under conditions that generate cell wall damage. Vancomycin is an antibiotic that binds to peptidoglycan precursors and prevents their cross-linking to the existing cell wall. While *E*. *coli* and most gram-negative bacteria are typically resistant to vancomycin, the *imp4213* allele in the *lptD* gene disrupts synthesis of the lipopolysaccharide component of the outer membrane, leading to a permeable outer membrane and increased sensitivity to vancomycin [29]. Our previous results showed that vancomycin treatment of *imp4213* cells leads to the formation of pores in the cell wall, from which the inner membrane and cytoplasm eventually escape when the pore size increases sufficiently [15]. Before the point of this blebbing, it is difficult to detect the level of damage using existing light microscopy techniques.

**Fig 3:**
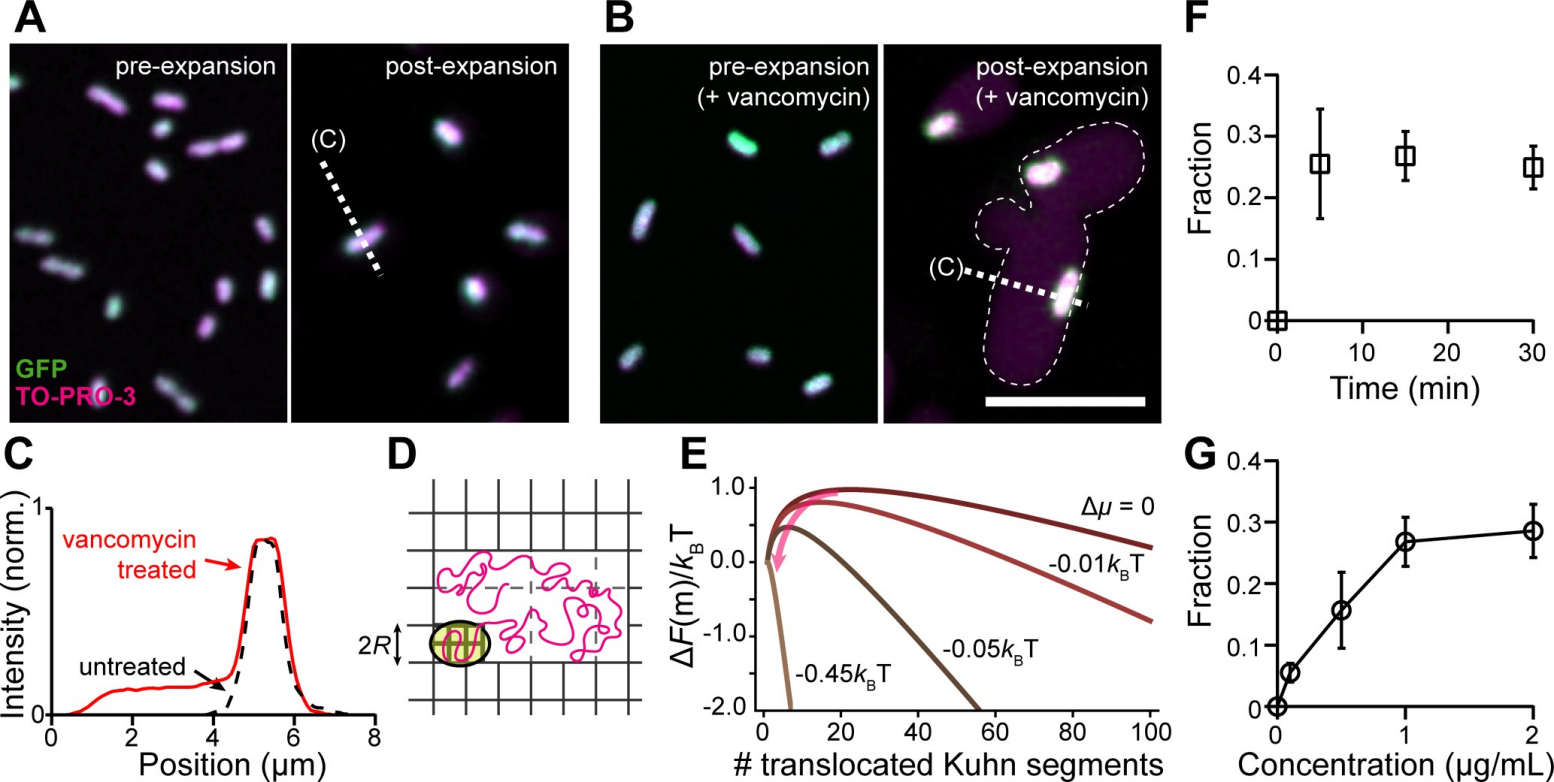
μExM detects cell wall damage induced by antibiotics with high sensitivity. (A) Images of GFP-expressing *imp4213 E*. *coli* cells, with DNA co-stained using TO-PRO-3, before (left) and after (right) expansion. (B) Images of vancomycin-treated *imp4213* cells, in which expansion leads to a large halo of DNA fluorescence surrounding the unexpanded cytoplasm. All images are maximum intensity projections. Scale bars, 10 μm. (C) Normalized intensity profiles of TO-PRO-3 fluorescence measured along dashed lines in (A) and (B). The data underlying this figure are included in S6 Data. (D) Proposed mechanism of DNA expansion via the translocation of a DNA chain. Yellow, cytoplasm; magenta, DNA; gray, gel network. Note the high-density gel network in the cell and low-density network around the cell. (E) The free energy for DNA translocation, Δ*F*(*m*), as a function of the *m*th Kuhn segment anchored at the pore. Δ*F*(*m*) has a maximum at m*, which presents an entropic barrier for DNA translocation. The lower density outside the cell wall due to expansion leads to a negative chemical potential difference (Δ*μ*), which facilitates the translocation process by reducing m* (arrow) and eventually causes the entropic barrier to vanish (Δ*μ*<−0.45*k*_*B*_*T*) for spontaneous DNA translocation. (F, G) Fractions of cells exhibiting an expanded DNA halo as a function of vancomycin treatment duration (concentration was fixed at 1 μg mL^−1^) (F) and concentration (treatment duration was fixed at 15 min) (G). Error bars represent SEM for three replicate experiments. The data underlying this figure are included in S7 Data. GFP, green fluorescent protein; μExM, expansion microscopy of microbes; norm., normalized; SEM, standard error of the mean.

We first treated *imp4213* cells for 10 min with 1 μg mL^−1^ vancomycin; at this early time point, cells did not exhibit any morphological changes due to drug treatment. Unlike untreated controls that remained unexpanded (**Fig 3A**), expansion of vancomycin-treated *imp4213* cells showed a striking pattern: surrounding the unexpanded cytoplasm (GFP labeled) was a halo of DNA (TO-PRO-3 labeled) that occupied a space with a width approximately 4 times that of an unexpanded cell (**Fig 3B and 3C**). We interpret this pattern as the translocation of DNA through pores in the cell wall during expansion (**Fig 3D**). As the hydrogel network contained within the cell wall is unable to expand with the surrounding network, an extracellular cavity with low-density networks is created that lowers the effective chemical potential and drives the DNA to spread into this cavity (**Fig 3E**). We estimate the minimum chemical potential difference required for the spontaneous translocation of *E*. *coli* DNA to be approximately 0.5 *k*_*B*_*T* (**Materials, Methods, and Models**), which is comparable to the thermal energy. This calculation indicates that DNA translocation is a sensitive measure of cell wall damage, as the DNA chain should always escape the confinement of the cell wall as long as the pore size grows to approximately 100 nm (the Kuhn length of DNA). The size of these pores would be below the diffraction limit and thus invisible using conventional optical approaches.

Using DNA translocation as the readout, we quantified the fraction of cells with damaged cell wall as a function of both vancomycin treatment time and concentration. Our results suggest that vancomycin-induced cell wall damage occurs more rapidly than previously appreciated [15]: the fraction of *imp4213* cells undergoing DNA translocation after expansion increased in the first 5 min of treatment and was approximately constant thereafter (**Fig 3F**). The level of cell wall damage detected by μExM was also concentration dependent, supporting the conclusion that the DNA translocation is a direct result of vancomycin treatment (**Fig 3H**). Interestingly, the fraction of cells showing DNA translocation plateaued at approximately 30%, indicating a heterogeneous response to vancomycin even at high concentrations. Overall, our results suggest that μExM can be used as a sensitive and quantitative assay to detect cell wall damage.

### μExM resolves bacterial species within a model animal gut microbiota

**Fig 4:**
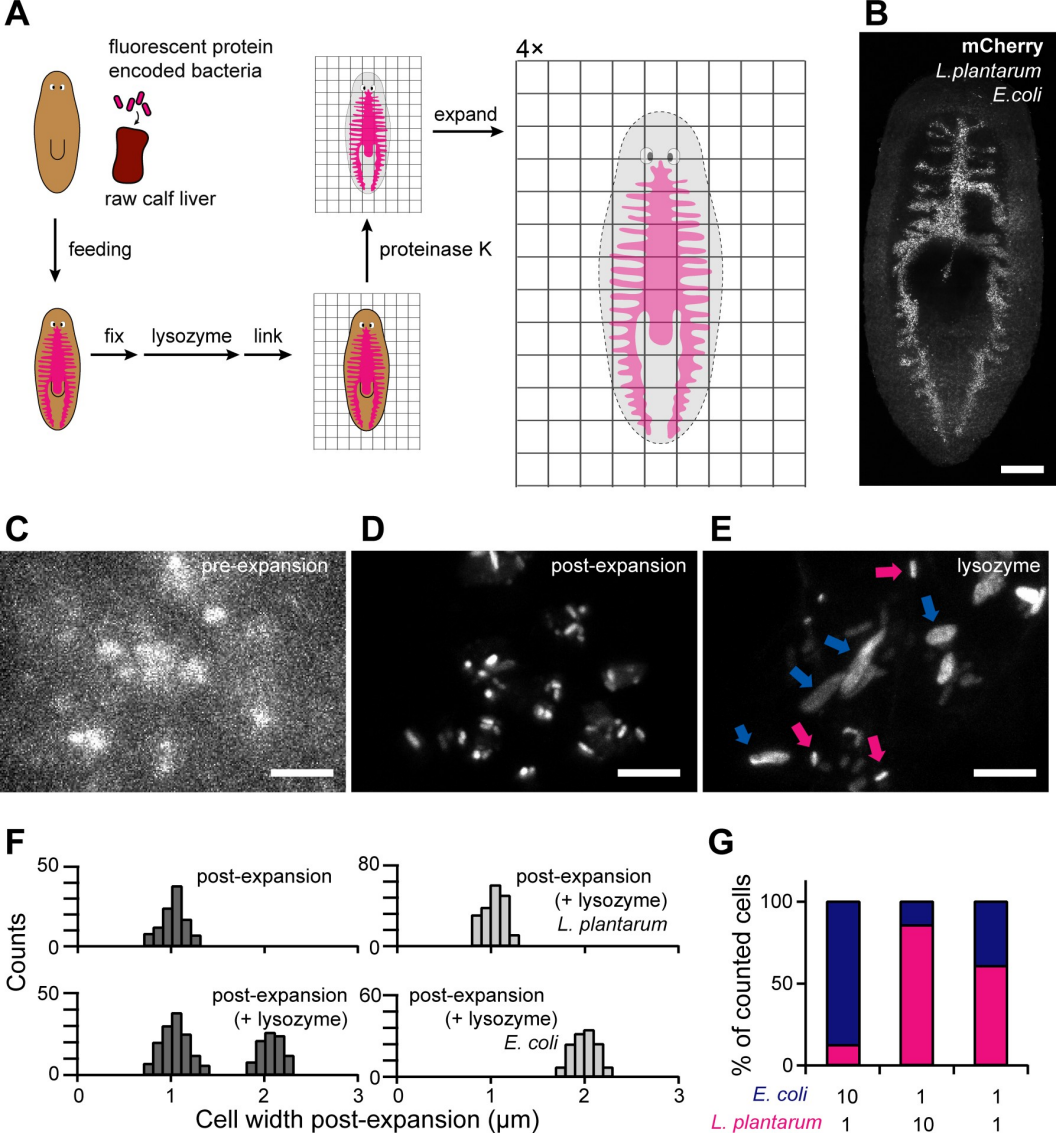
μExM resolves different bacterial species in the planarian flatworm gut. (A) Schematic of the μExM workflow for planarians. Planarians were fed with fluorescent bacteria, and fixed. Unlike other ExM protocols, μExM uses lysozyme or mutanolysin to digest the bacterial cell wall. Linker molecules were then used to anchor the planarian tissue as well as microbial proteins to the hydrogel network. After digestion with proteinase K, the hydrogel was expanded 4-fold isotropically. (B) Pre-expansion maximum-intensity projection of a planarian with its gut colonized by a mixture of *E*. *coli* and *L*. *plantarum*, both expressing mCherry. Imaging was performed 3 d after feeding the planarian with microbes. Scale bar, 200 μm. (C–E) Magnified views showing microbial populations before expansion (C), after expansion (D), and after expansion with lysozyme treatment (E). In (E), magenta arrows indicate unexpanded cells (*L*. *plantarum*) and blue arrows indicate expanded cells (*E*. *coli*). Scale bars, 10 μm. (F) Quantification of cell width of the mixed populations of *E*. *coli* and *L*. *plantarum* in the planarian gut (left). Right, in vivo control populations containing a single species. The data underlying this figure are included in S8 Data. (G) Species composition in the planarian gut at 3 d post-feeding, counted based on cell width after lysozyme treatment and expansion. *n* > 250 cells were measured for each condition. The relative abundance of the two species in the initial mixture fed to the planarians is shown below the plot. ExM, expansion microscopy; μExM, expansion microscopy of microbes.

**Fig 5:**
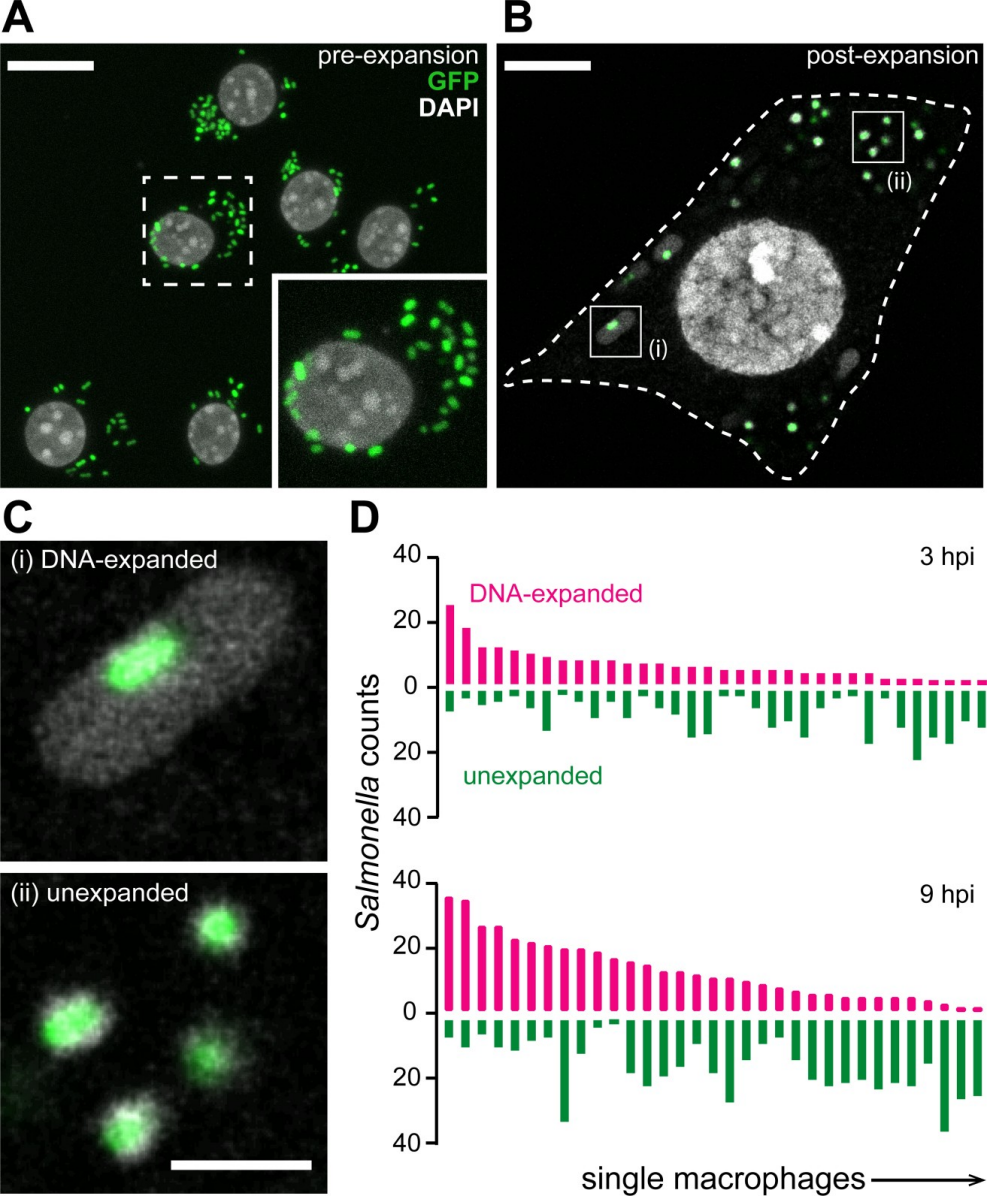
μExM detects changes in the cell wall structure of macrophage-engulfed *Salmonella* cells. (A, B) Confocal images of RAW264.7 cells infected with GFP-*Salmonella* 3 h postinfection before (A) and after (B) expansion. Inset in (A), magnified view of the dashed box. Dashed line in (B), macrophage periphery. Scale bars, 20 μm. (C) Magnified views showing two populations of *Salmonella*: DNA-expanded (top) and unexpanded (bottom), corresponding to cells highlighted by the boxes in (B). Scale bar, 5 μm. (D) Number of expanded and unexpanded *Salmonella* cells in individual macrophages determined by manual counting at 3 h (top) and 9 h (bottom) postinfection. Note that the numbers of both types increase with time as *Salmonella* cells proliferate. The data underlying this figure are included in S9 Data. GFP, green fluorescent protein; hpi, hours post-infection; μExM, expansion microscopy of microbes.

To demonstrate the utility of expansion as an imaging contrast, we focused on two applications: (1) in vivo imaging to resolve bacterial species in an animal gut, particularly when strain-specific fluorescent tags may not be available or are limited by host tissue autofluorescence (**Fig 4**), and (2) detection of cell wall disruption in situ when pathogenic bacteria are under attack from host defense mechanisms (**Fig 5**). In both cases, traditional spectral contrast in fluorescence microscopy would be insufficient, while μExM reveals new biological insights.

First, we colonized the gut of a model organism, the planarian flatworm *Schmidtea mediterranea* [30,31], with *E*. *coli* and *L*. *plantarum*, both expressing mCherry (**S2 Fig**). Use of mCherry was prudent as planarian tissues have strong autofluorescence below 560 nm, limiting the utility of other fluorescent proteins such as GFP or YFP that spectrally overlap with the autofluorescence (**S3 Fig**). The bacteria were introduced by feeding the planarian with a calf liver–bacteria mixture (**Materials, Methods, and Models**), after which colonization was allowed to stabilize for 3 d (**Fig 4A**). The planarians were then fixed and imaged using the μExM protocol optimized for planarian tissues (**S4 Fig**).

Before expansion, bacterial cells in the planarian gut were barely resolvable (**Fig 4B and 4C**). Expansion clearly revealed the borders of individual cells, as distances between cells increased (**Fig 4D** and **S4 Fig**) and the optical clearing of planarian tissues improved the signal-to-noise ratio (**S4 Fig**). Moreover, the two species became distinguishable after lysozyme treatment and expansion (**Fig 4E**); individual cell widths split into two populations corresponding to *E*. *coli* (approximately 2-fold expanded) and *L*. *plantarum* (mostly unexpanded) (**Fig 4F**, left). Single-species in vivo controls verified that there is little to no overlap between the two populations (**Fig 4F**, right). While expansion ratios were robust across replicate experiments, we noted secondary but statistically significant (*p* < 0.0001) differences in expansion ratios of *E*. *coli* cells between our in vitro (mean ± SEM, 2.21 ± 0.04) and in vivo (2.06 ± 0.01) measurements, supporting the idea that the environmental factors (e.g., pH, temperature, oxygen level, etc.) in the planarian gut may induce changes in cell wall structure during bacterial colonization. Nonetheless, this difference is small relative to the interspecies comparisons. We quantified the relative abundances of the two species early during colonization and found that they correlated well with those of the initial mixture fed to the planarians (**Fig 4G**). Together, this application demonstrates that μExM can provide quantitative measures of the species composition of defined gut microbiotas, a critical step toward resolving the key factors that determine compositional dynamics [24,32].

### μExM reveals previously unrecognized cell-to-cell phenotypic heterogeneity among pathogenic bacteria during infection

Next, we investigated *Salmonella* cells during macrophage infection. The fate of *Salmonella* after entering macrophages is known to be heterogeneous: some cells survive and proliferate, whereas others lyse in the harsh intracellular environment [33–35]. Previous studies have suggested that variations in *Salmonella* cell wall structure may play an important role in heterogeneous infection outcomes [36], but it has been challenging to measure such phenotypic variations in situ.

We used μExM to image GFP-*Salmonella* cells engulfed by RAW264.7 macrophages, co-staining DNA with DAPI. We observed two types of heterogeneity. First, expansion of individual *Salmonella* cells exhibited two distinct states (**Fig 5A and 5B**): some cells remained unexpanded, indicative of an intact cell wall consistent with our in vitro experiments (**S5 Fig**), whereas others exhibited an expansion pattern similar to *E*. *coli imp4213* cells after vancomycin treatment (**Fig 3**), suggesting the presence of submicrometer pores in the cell wall, through which DNA escaped during expansion to form a halo around the unexpanded cytoplasm (**Fig 5C**). Second, the fraction of DNA-expanded *Salmonella* cells varied drastically between individual macrophages (**Fig 5D**). The observed heterogeneity was consistent across time points postinfection. These observations reveal stochasticity in the fate of *Salmonella* cells during macrophage infection.

## Discussion

Here, we develop an ExM method (μExM) for bacteria and demonstrate that expansion patterns are determined by cell wall structural properties. We use this phenomenon as a nonconventional imaging contrast and demonstrate the utility of μExM via in vivo imaging of gut microbial communities and detection of cell-to-cell heterogeneity among pathogenic bacteria as they infect macrophages. We expect this method to spur new research in three major areas.

First, we have shown that in order to be applicable to microbiology, ExM must be modified to predigest the bacterial cell wall with specific enzymes such as mutanolysin to achieve full, uniform expansion. It has been demonstrated that ExM is compatible with conventional antibodies [21,25,37] and RNA FISH [20]; therefore, μExM is readily adaptable to image nanometer-scale ultrastructures in bacterial cells that are under the diffraction limit of optical imaging. Subcellular organization in bacterial cells is a field of active discovery [38], but super-resolution information has thus far been accessible only through specialized equipment [22,39,40]. μExM should open up new applications and provide technical convenience in this burgeoning research area. Moreover, recent progress [41, 42] has integrated ExM with other super-resolution imaging techniques (e.g., stimulated emission depletion microscopy [STED], structured illumination microscopy [SIM]) for further improved “ultra” resolution, suggesting μExM may have the opportunity to overcome current resolution limits in imaging bacterial ultrastructures.

More importantly, beyond the established strengths of ExM (i.e., improved spatial resolution and high signal-to-noise ratio), the differential expansion between cells with partially digested cell walls offers a new imaging contrast that is orthogonal to spectral separation in standard fluorescence microscopy using FISH probes, chemical modification, antibodies, or fluorescent proteins. Currently, many bacterial species are considered genetically intractable and hence cannot be transformed for strain-specific labeling, while FISH experiments are often technically challenging. With three relevant applications (in vitro communities of human commensal bacteria, in vivo gut imaging, and macrophage infection), we have demonstrated that the contrast in expansion is quantitative and sensitive enough to resolve cells of different species in a mixed population or in distinct physiological states, which are otherwise difficult to capture using traditional imaging methods. As μExM does not require any special microscopy instrumentation, it can be easily integrated with other optical methods for high-content multimodal imaging. For example, combining expansion and spectral labeling may enable concurrent tracking of dozens of microbial species in a complex community or detecting intermediate states as cells undergo physiological changes. We thus anticipate μExM will have broad applications in studies of complex bacterial communities in microbiota, biofilms, and at host–microbe interfaces.

Finally, while cell wall stiffness can be measured using direct mechanical methods (e.g., atomic force microscopy [43] or Brillouin microscopy [44]), these methods are not applicable to cells in dense populations and in vivo conditions. μExM can quantitatively evaluate cellular phenotypes in vivo and in situ under various genetic, chemical, or physical perturbations. These perturbations can include, for instance, genetic disruption of cell wall synthesis or chemical stresses such as antibiotic treatment [15,45], pH changes, and osmotic shock. μExM phenotypes depend on the rupture point of the cell envelope, which is highly relevant to perturbations meant to disrupt the mechanical integrity of bacterial cells such as antibiotics. Future investigations should highlight the power of μExM for comparing cell wall phenotypes both in isolated cells and within a dense, complex community. With the recent discovery of microscale spatial organization in the human microbiota [4,23], μExM will be a powerful tool for revealing how spatial neighborhoods modulate cellular phenotypes.

## Materials, methods, and models

### Bacterial sample preparation

Bacterial strains and culture conditions used in this study are summarized in **Table 1**. For in vitro samples, cells were collected from overnight cultures via centrifugation at 2,000*g* for 5 min, washed twice in PBS, and fixed in PBS containing 4% formaldehyde and 1% NP-40 for 10 min. After fixation, cells were washed in PBS and then resuspended in PBST (PBS supplemented with 0.3% Triton X-100) for 30 min at room temperature. As optical density (OD) is unreliable in determining bacterial number densities across species, we quantified relative cell number densities through spreading 10 μL of DAPI-stained suspension between a coverslip and a glass slide and counting cells in 5 images (field of view = 440 μm × 330 μm) using epifluorescence microscopy. The resuspended bacteria were sequentially dehydrated in 50:50% methanol:PBST and then pure methanol to remove lipids. Dehydrated cells can be kept at −20°C for several months.

For vancomycin treatment, *E*. *coli imp4213* cells were grown to early stationary phase (OD_600_ = 0.8) and incubated in media containing vancomycin (Sigma-Aldrich, St. Louis, MO) at 37°C before fixation. We focused on cells during the early stationary phase because we noticed large cell-to-cell variations in expansion between dividing cells during exponential growth.

### CFU measurements of bacterial cocultures

To quantify relative bacterial abundances, serial dilutions of cocultures were plated on two different media to count the CFUs of individual species in the mixture. *Citrobacter* sp. and *B*. *ovatus* were enumerated by plating on GAM supplemented with menadione and hemin and growing for 2 d at 37°C anaerobically. *B*. *ovatus* was differentiated from *Citrobacter* sp. on GAM by its formation of small pinprick colonies. *B*. *breve* was counted by plating the coculture on MRS media and growing for 2 d at 37°C anaerobically. *B*. *breve* was distinguished from *Citrobacter* sp. on MRS by its formation of opaque white colonies.

### Planarian sample preparation

Asexual *S*. *mediterranea* planarians were maintained at 20°C in ultrapure water supplemented with 0.5 g L^−1^ Instant Ocean salts and 0.1 g L^−1^ NaHCO_3_ and were fed calf liver paste once or twice weekly. To colonize the planarian gut with bacteria, planarians were starved for at least 7 d, then fed with 250 μL calf liver paste mixed with 50 μL of a mixture of *L*. *plantarum* and *E*. *coli* cells, which were collected from 5-mL cultures in early stationary phase (OD_600_ = 0.8–1.0) and concentrated in 1 mL of PBS. After 3 d, individual planarians were collected into separate tubes and fixed individually (to avoid clumping) in PBST containing 4% formaldehyde and 1% NP-40 for 2 h at room temperature. The fixed planarians were washed in PBST, dehydrated in 50:50% methanol:PBST followed by pure methanol, and stored at −20°C.

To label muscle fibers using immunofluorescence, planarians were killed by 2% HCl for 5 min and then fixed in 4% formaldehyde with 1% NP-40 for 2 h at room temperature. Samples were rinsed briefly in PBST and bleached overnight at room temperature with 6% H_2_O_2_ in PBST under bright light. The planarians were rinsed with PBST, blocked in PBST supplemented with 1% (w/v) BSA (PBSTB) for 4 h at room temperature, and then incubated with the antibody 6G10 (DSHB, 1:1,000 dilutions in PBSTB) for 12–15 h at 4°C [46]. At least 6 washes of 20 min each with PBST were carried out prior to adding the peroxidase-conjugated secondary anti-mouse antibody (Jackson ImmunoResearch, West Grove, PA) at a 1:1,000 dilution in PBSTB. After overnight incubation at 4°C, samples were extensively washed in PBSTB. Tyramide signal amplification was performed by incubating planarians for 10 min in homemade TAMRA-conjugated tyramide in 100 mM borate buffer (pH = 8.5) supplemented with 2 M NaCl, 0.003% H_2_O_2_, and 20 μg mL^−1^ 4-iodophenylboronic acid.

### Macrophage sample preparation

To infect macrophages with *Salmonella*, RAW264.7 cells were plated at 500,000 cells/well on coverslips coated with fibronectin (10 μg mL^−1^ for 30 min) in 6-well plates and allowed to attach overnight. Cells were rinsed three times with Fluorobrite DMEM media (ThermoFisher Scientific, Waltham, MA) supplemented with 10 mM HEPES, 1% FBS, and 2 mM L-glutamine. Overnight cultures of *Salmonella* were diluted in Fluorobrite DMEM media and added to wells at a 1,250:1 multiplicity of infection (MOI). The plate was centrifuged (200*g*) for 15 min at 34°C. Infected macrophages were washed twice to remove extra bacteria, and 1 mL of medium containing 10 μg mL^−1^ gentamicin was added to each well. Cells were cultured at 37°C under 5% CO_2_ for 3–9 h, then fixed in PBS containing 4% formaldehyde and 1% NP-40 for 10 min. After fixation, cells were dehydrated in methanol and stored at −20°C.

### μExM

Catalog numbers of all reagents are provided in **S1 Table**. Dehydrated samples (bacterial cells, planarian tissues, infected macrophages) were kept at −20°C for at least overnight and sequentially rehydrated with 50:50% methanol:PBST, then PBST. To digest bacterial cell walls, samples were incubated overnight at 37°C in either PBS containing 0.02–2 mg mL^−1^ lysozyme (ThermoFisher Scientific, Waltham, MA) or in 50 mM phosphate buffer (pH = 4.9) containing 160 units (U) mL^−1^ mutanolysin (Sigma-Aldrich, St. Louis, MO) [47], unless otherwise specified.

After cell wall digestion, the μExM protocol follows a sequence of gelation, proteinase K digestion, and expansion, as previously described [25]. Briefly, samples were rinsed three times with PBS, then incubated for 1 h in a PBS solution of 1 mM methacrylic acid *N*-hydroxysuccinimide ester (MA-NHS; Sigma-Aldrich, St. Louis, MO), freshly diluted from a 1 M MA-NHS stock in DMSO. After rigorous washes with PBS, bacterial cells were collected through centrifugation and resuspended in monomer solution (1× PBS, 2 M NaCl, 8.625% [w/w] sodium acrylate, 2.5% [w/w] acrylamide, 0.15% [w/w] N,N′-methylenebisacrylamide) for 1 min before gelation. Similarly, macrophages on coverslips and planarian tissue were incubated in the monomer solution for 1 min and 45 min at 4°C before gelation, respectively.

Gelation was performed in chambers that were assembled using #1.5 coverslips as spacers placed between microscope slides. Gelation was initiated by adding ammonium persulfate stock (10% [w/w] in monomer solution, ThermoFisher Scientific, Waltham, MA) and tetramethylethylenediamine (10% [w/w] in monomer solution, ThermoFisher Scientific, Waltham, MA) to the final concentration of 0.2% (w/w). For planarian tissues and macrophage samples, 4-hydroxy-2,2,6,6-tetramethylpiperidin-1-oxyl (4-hydroxy-TEMPO, Sigma-Aldrich, St. Louis, MO) was added from a 0.5% (w/w) stock solution to a final concentration of 0.01% (w/w) to inhibit gelation during the diffusion of monomers into tissues. Gelation was completed by incubation at 37°C for 1–2 h. During this process, bacterial cells typically sedimented to the bottom of the gel.

After gelation, excess gel around tissue samples was removed, and then gelled samples were gently removed from the chamber and digested overnight at 37°C in 8 U mL^−1^ proteinase K (NEB, Ipswich, MA) diluted in digestion buffer (1× TAE buffer, 0.5% Triton X-100, 0.8 M guanidine HCl). Gels were then removed from digestion buffer and placed in excess Milli-Q water to expand. Water was exchanged every 15 min 3–5 times until the size of the expanded gels plateaued. To stain DNA, expanded samples were incubated with 100 μM DAPI or 1 μM TO-PRO-3 for 30 min.

Fluorescence confocal imaging was performed on a Zeiss LSM 800 using either a 20× (N.A. = 1.0, working distance = 1.8 mm) water-immersion objective (W Plan-Apochromat) or a 40× (N.A. = 1.1, working distance = 0.62 mm) water-immersion objective (LD C-Apochromat Corr M27). Expanded samples were mounted in imaging chambers assembled from iSpacer (3.0-mm deep, Sunjin lab, Hsinchu City, Taiwan) sandwiched between two coverslips. To avoid lateral drift during image acquisition, the expanded gels were immobilized on the coverslip using a small amount of epoxy adhesive (3 M, Maplewood, MN) applied around the gel. To image a large area, tiled images were stitched using either Zen (Zeiss, Oberkochen, Germany) or FIJI software. Maximum intensity projection images were generated using built-in functions. The FIJI plugin “MorphoLibJ” was used for the morphological segmentation of bacterial cells [48]. After segmentation, a custom MATLAB script was used to measure aspect ratio and cell width. Cell width was computed along the short axis and averaged at five locations evenly spaced along the long axis.

### Estimate of chemical potential for spontaneous translocation of DNA from cell wall confinement

We model the translocation of a DNA chain through a pore in the cell wall to consist of two primary steps. First, we assume that one end of the DNA is anchored near the pore. The anchoring energy and the loss of conformational entropy due to the localization of the chain end give rise to a free energy barrier of the form [49]

$$\frac{F^{†}}{k_{B}T}=\frac{\varepsilon }{k_{B}T}+\ln(\frac{4R^{4}}{\pi av}),$$

where *ε* is the anchoring energy, *R* is the confinement radius (essentially equivalent to the radius of the cell), *a* is the range of anchoring near the pore, and *v* is the volume of the anchored segment. This free energy barrier must be overcome to initialize translocation. Because the anchoring energy depends on the details of the pore and the way it interacts with the anchored segment, it is impractical to evaluate exactly the numerical value of this barrier. Nonetheless, it is apparent that stronger confinement (smaller *R*) lowers the barrier height. Moreover, the presence of multiple pores in the cell wall that are sufficiently large for translocation increases the probability that the DNA is anchored at such a pore.

The second step concerns the actual translocation. After anchoring, the DNA chain diffuses along its backbone, outwards or inwards, across the pore. At any instant, one particular segment (labeled *m*) is anchored at the pore, reducing the conformational entropy of the DNA chain (**Fig 2D**). By treating the chain as a Gaussian random walk, the free energy associated with this entropy loss can be obtained in terms of a series summation [50]. In the limit of strong confinement (i.e., when *R* is smaller than the radius of gyration of the DNA, *R*_*g*_), the ground-state dominance approximation [49] leads to the following expression for the free energy as a function of *m*:

$$\frac{F(m)}{k_{B}T}=-\ln\left[1+\frac{R}{R_{g}}{\left(\frac{N}{\pi m}\right)}^{\frac{1}{2}}\right]+\frac{\pi^{2}(N-m)}{N}{\left(\frac{R_{g}}{R}\right)}^{2}+m\frac{\Delta \mu }{k_{B}T},$$

where *N* is the total number of segments and Δ*μ* is the difference in chemical potential of each segment outside and inside the cell. The first and second terms come from the conformational entropies of the two half-chains outside and inside the cell, respectively. Note that the free energy depends on the confinement through the ratio *R*/*R*_g_.

We evaluated the free energy difference Δ*F*(*m*) ≡ *F*(*m*)−*F*(1) (**Fig 2E**) for the nucleoid confined inside a bacterial cell. We set the radius of the cell to be *R* = 1 *μm* and the length of DNA to be that of the *E*. *coli* genome, which is 4.7 Mbp giving a contour length *L* of 1.596 mm. Using 1 nm as the radius of the cross section, we estimate that the DNA fills approximately 0.1% of the intracellular volume, and hence crowding should not substantially hinder segmental motions. Estimating the Kuhn length of DNA at *l*_*K*_ = 100 *nm*, we find the number of Kuhn segments *N* = *L*/*l*_*K*_ = 15,980 and the radius of gyration *R*_*g*_ = *N*^1/2^*l*_*k*_ = 5.2 *μm*. As a result, the DNA is strongly confined, with *R*/*R*_*g*_ = 0.19.

The salient feature of our model across chemical potential differences Δ*μ* is the existence of a single, entropic barrier for molecular translocation (**Fig 2E**). This barrier originates from the entropy loss associated with translocating the first few segments outside the pore, in addition to those originally anchored inside. Once this entropic barrier is overcome, the free energy decays monotonically with *m*, and DNA translocation proceeds spontaneously. Differentiating the free energy leads to a cubic equation for the location of the barrier, m*, which shows that m* depends on the confinement size *R*/*l*_K_ and on the chemical potential difference Δ*μ*, but not on the DNA length *N*. In particular, for Δ*μ* = 0, m* = 0.23(*R*/*l*_*k*_)^2^; in this case, for *R* = 1 *μm*, m* = 23, indicating that 23 Kuhn segments (equivalent to approximately 6.7 kbp) need to be successfully translocated via diffusive motion along the chain contour before the whole chain spontaneously escapes the confinement.

Lowering the segmental chemical potential outside the cell reduces m* and lowers the height of the barrier (**Fig 2E**). As external localization becomes sufficiently friendly (lower Δ*μ*), the entropic barrier may disappear altogether. By setting m* = 1, we identified that such a transition occurs when

$$\Delta \mu \leq \Delta \mu_{c}=\left[\frac{\pi }{{z_{c}}^{2}}-\frac{z_{c}}{2(1+z_{c})}\right]k_{B}T,$$

where $z_{c}=\sqrt{\frac{6}{\pi }}\frac{R}{l_{K}}$. For *R*/*l*_*k*_ = 10, Δ*μ*_*c*_ = −0.45*k*_*B*_*T* is the chemical potential at which the energy barrier for DNA translocation vanishes.

## Supporting information

S1 Fig μExM expands microbial species to different extents. (A) Representative μExM images of mCherry–*E*. *coli*. Corresponding distributions of cell widths with the various treatments are shown in **Fig 1B**. (B) μExM images of *A*. *intestini*, with DNA stained using TO-PRO-3. Corresponding distributions of cell widths are shown in **Fig 1B**. (C) Representative μExM images of various human commensal bacterial species. DNA was stained with TO-PRO-3. Blue, pre-expansion images; magenta, post-expansion images after lysozyme treatment. Corresponding distributions of cell widths are shown below the images. The data underlying this figure are included in S10 Data. All images are maximum intensity projections. Scale bars, 10 μm. μExM, expansion microscopy of microbes.
(TIF)

S2 Fig Imaging of fluorescently labeled bacteria colonizing the planarian gut. (A) Confocal image showing a whole planarian fed with mCherry–*E*. *coli* at 3 d post-feeding. The planarian gut and its branches (dotted line) are clearly visible. Scale bar, 500 μm. (B–D) Transverse sections of the planarian trunk region showing that mCherry–*E*. *coli* are primarily located inside the planarian gut. Dashed line: the outline of the planarian body. Scale bars, 200 μm. (E) A representative section of the planarian gut colonized by mCherry–*L*. *plantarum* at 3 d post-feeding. Dashed line: the outline of gut branches. (F, G) Magnified views of the highlighted region (dashed red square) in (E), before expansion (F) and after expansion (G). Without cell wall digestion, *L*. *plantarum* cells remained unexpanded, but the distances between cells increased 4-fold, allowing single cells to be optically resolved. All images are maximum intensity projections. Scale bars, 10 μm. A, anterior; D, dorsal; P, posterior; ph, pharynx; V, ventral.
(TIF)

S3 Fig Autofluorescence of planarian tissue. Epifluorescence images showing the strong autofluorescence exhibited by planarian tissues at wavelengths below 560 nm. Arrowheads highlight planarian eye spots, which are visible at shorter wavelengths. Scale bars, 50 μm.
(TIF)

S4 Fig Optimization of μExM for planarian tissues. (A–C) Tissue clearing by digestion and expansion. Grids in the background were included to show tissue transparency. Dashed lines in (C): the outline of the planarian body, which is larger than the imaging view. Scale bars, 1 mm. (D, E) ExM of planarian tissues following a protocol similar to [31], but using a different linker molecule. While the previous study [31] used 6-((acryloyl)amino)hexanoic acid, succinimidyl ester (acryloyl-X, SE) as the linker, we tested glutaraldehyde (GA) (D) or MA-NHS (E) as linker molecules. Post-expansion images of planarians immunostained for muscle fibers demonstrated that expansion using GA disrupts muscle fibers, whereas no distortion was observed in MA-NHS–linked tissues. Scale bars, 20 μm. acryloyl-X, SE, 6-((acryloyl)amino)hexanoic acid, succinimidyl ester; ExM, expansion microscopy; GA, glutaraldehyde; MA-NHS, methacrylic acid *N*-hydroxysuccinimide ester; μExM, expansion microscopy of microbes.
(TIF)

S5 Fig μExM of *S*. *enterica* cells in vitro. (A) Representative maximum intensity projection of mCherry-*Salmonella* cells before expansion. (B) After 1 h of lysozyme treatment to digest the cell wall, *Salmonella* cells expanded approximately 2-fold. Note that mCherry (left) and DAPI (right) signals colocalized. (C) Quantification of the expansion of cells in images similar to (B). The data underlying this figure are included in S11 Data. (D, E) Live cells that were treated with 0.5 mg mL^−1^ lysozyme for 1 h at 37°C prior to fixation (D) or cultured in an acidic, magnesium-depleted minimal medium (MgM-MES, pH 5.0, used to mimic the low pH, low Mg^2+^ environment of the phagosome) (E) did not expand, indicating that the cell wall remained intact under these conditions. Scale bars, 10 μm. MgM-MES, magnesium minimal MES medium; μExM, expansion microscopy of microbes.
(TIF)

S1 Table Reagents used in μExM. μExM, expansion microscopy of microbes.
(DOCX)

S1 Data Raw data of Fig 1B. (XLSX)

S2 Data Raw data of Fig 1E. (XLSX)

S3 Data Raw data of Fig 2B. (XLSX)

S4 Data Raw data of Fig 2C. (XLSX)

S5 Data Raw data of Fig 2D. (XLSX)

S6 Data Raw data of Fig 3C. (XLSX)

S7 Data Raw data of Fig 3F and 3G. (XLSX)

S8 Data Raw data of Fig 4F. (XLSX)

S9 Data Raw data of Fig 5D. (XLSX)

S10 Data Raw data of S1C Fig. (XLSX)

S11 Data Raw data of S5C Fig. (XLSX)

## Abbreviations

CFU: colony-forming unit
ExM: expansion microscopy
FISH: fluorescence in situ hybridization
GFP: green fluorescent protein
MA-NHS: methacrylic acid *N*-hydroxysuccinimide ester
μExM: expansion microscopy of microbes
MOI: multiplicity of infection
OD: optical density
SIM: structured illumination microscopy
STED: stimulated emission depletion microscopy
