# Supplementary figures and images for: Mechanically resolved imaging of bacteria using expansion microscopy
Source: PLoS Biol. 2019 Oct 17;17(10):e3000268. doi: 10.1371/journal.pbio.3000268 (PMC6797083; doi:10.1371/journal.pbio.3000268)

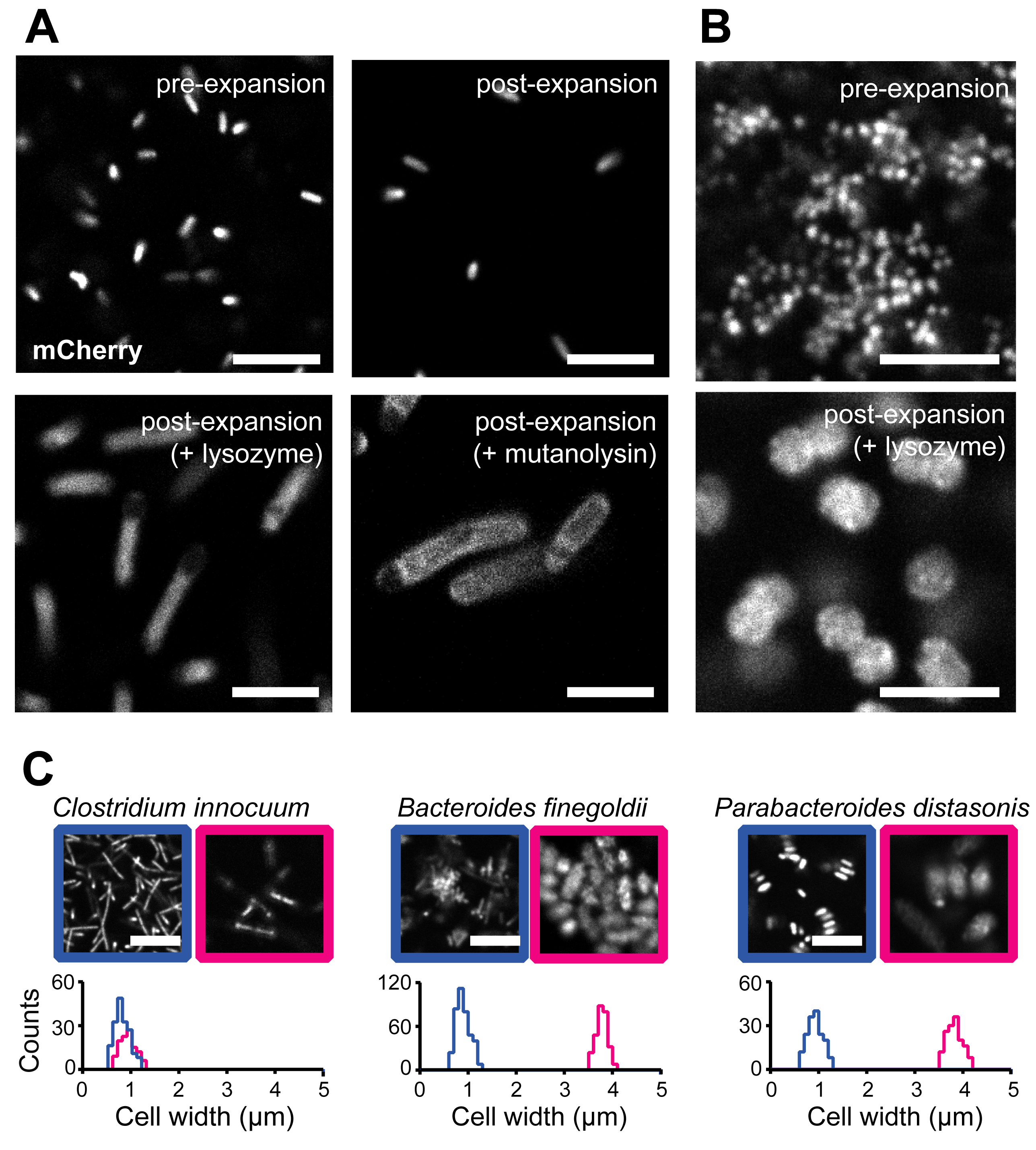

Supplement: S1 Fig — (A) Representative μExM images of mCherry–E. coli. Corresponding distributions of cell widths with the various treatments are shown in Fig 1B. (B) μExM images of A. intestini, with DNA stained using TO-PRO-3. Corresponding distributions of cell widths are shown in Fig 1B. (C) Representative μExM images of various human commensal bacterial species. DNA was stained with TO-PRO-3. Blue, pre-expansion images; magenta, post-expansion images after lysozyme treatment. Corresponding distributions of cell widths are shown below the images. The data underlying this figure are included in S10 Data. All images are maximum intensity projections. Scale bars, 10 μm. μExM, expansion microscopy of microbes. (TIF) [file pbio.3000268.s001.tif]

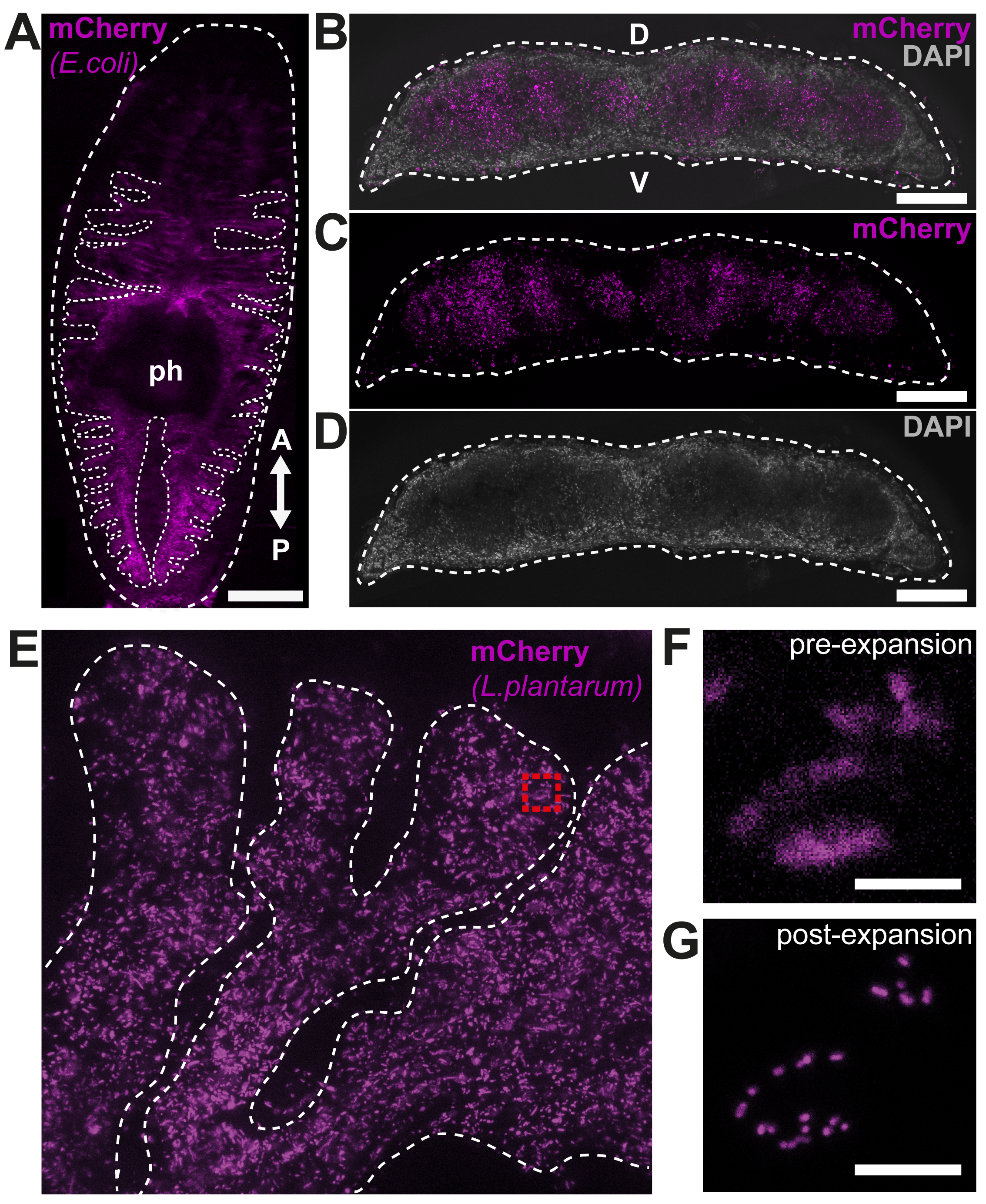

Supplement: S2 Fig — (A) Confocal image showing a whole planarian fed with mCherry–E. coli at 3 d post-feeding. The planarian gut and its branches (dotted line) are clearly visible. Scale bar, 500 μm. (B–D) Transverse sections of the planarian trunk region showing that mCherry–E. coli are primarily located inside the planarian gut. Dashed line: the outline of the planarian body. Scale bars, 200 μm. (E) A representative section of the planarian gut colonized by mCherry–L. plantarum at 3 d post-feeding. Dashed line: the outline of gut branches. (F, G) Magnified views of the highlighted region (dashed red square) in (E), before expansion (F) and after expansion (G). Without cell wall digestion, L. plantarum cells remained unexpanded, but the distances between cells increased 4-fold, allowing single cells to be optically resolved. All images are maximum intensity projections. Scale bars, 10 μm. A, anterior; D, dorsal; P, posterior; ph, pharynx; V, ventral. (TIF) [file pbio.3000268.s002.tif]

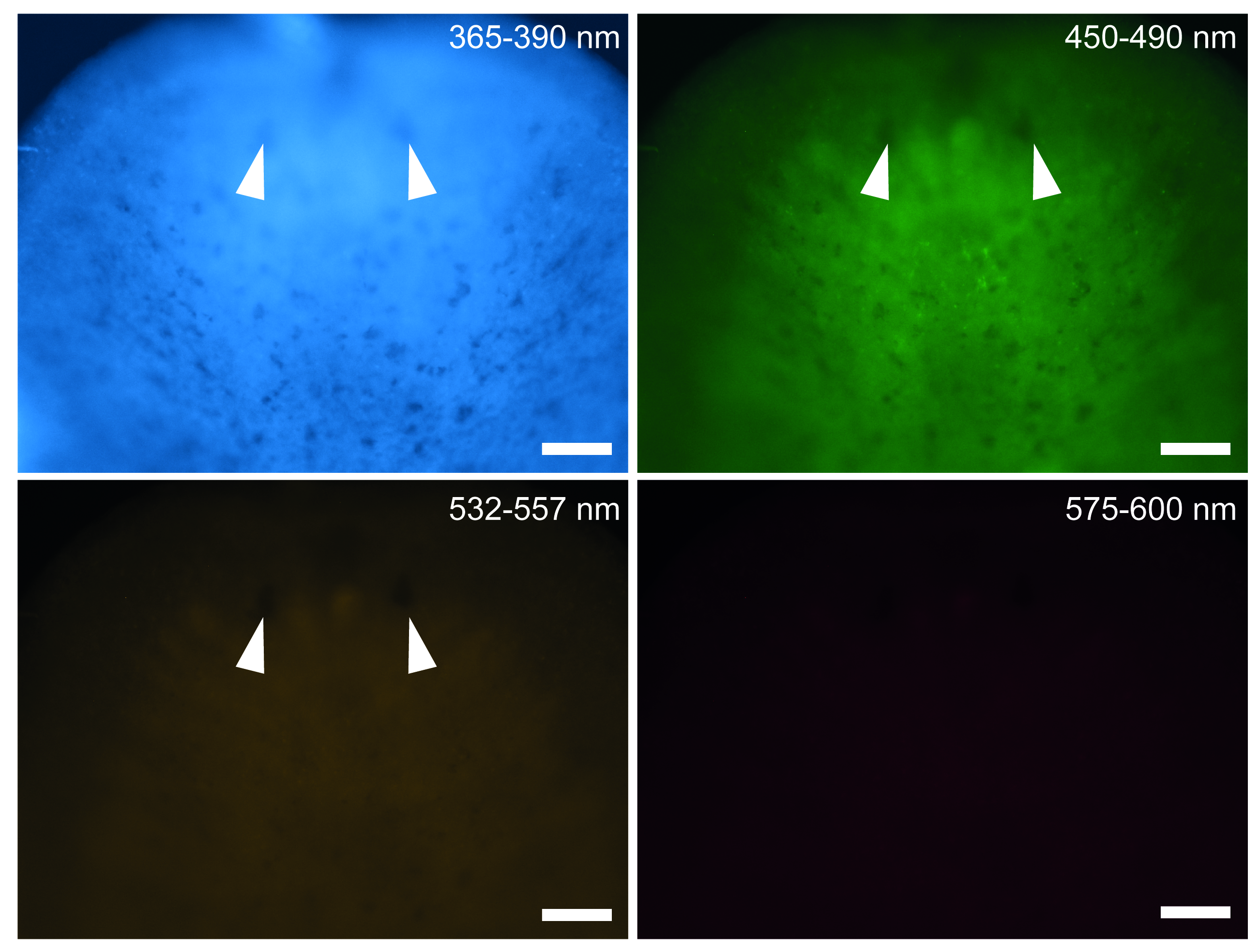

Supplement: S3 Fig — Epifluorescence images showing the strong autofluorescence exhibited by planarian tissues at wavelengths below 560 nm. Arrowheads highlight planarian eye spots, which are visible at shorter wavelengths. Scale bars, 50 μm. (TIF) [file pbio.3000268.s003.tif]

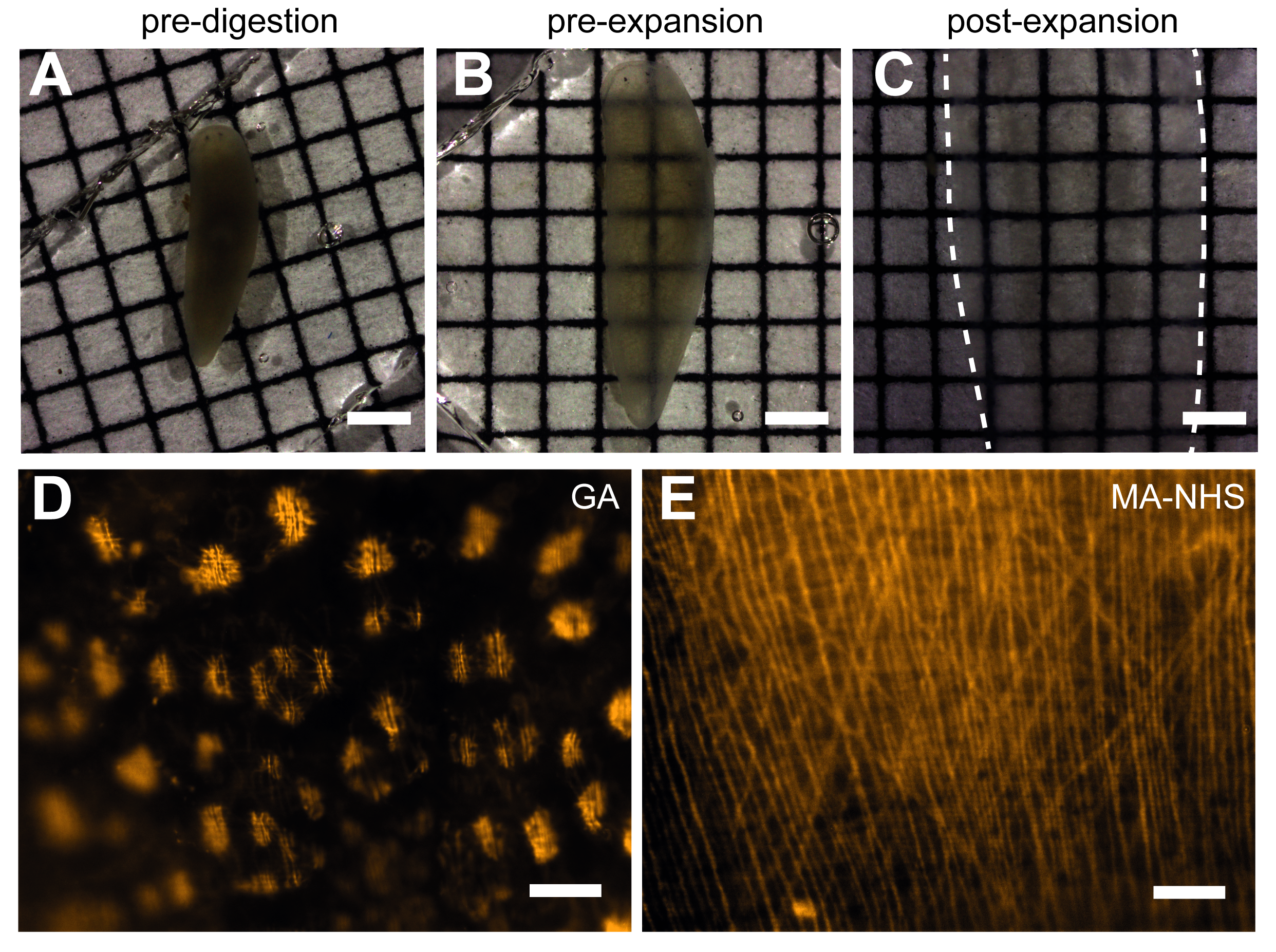

Supplement: S4 Fig — (A–C) Tissue clearing by digestion and expansion. Grids in the background were included to show tissue transparency. Dashed lines in (C): the outline of the planarian body, which is larger than the imaging view. Scale bars, 1 mm. (D, E) ExM of planarian tissues following a protocol similar to [31], but using a different linker molecule. While the previous study [31] used 6-((acryloyl)amino)hexanoic acid, succinimidyl ester (acryloyl-X, SE) as the linker, we tested glutaraldehyde (GA) (D) or MA-NHS (E) as linker molecules. Post-expansion images of planarians immunostained for muscle fibers demonstrated that expansion using GA disrupts muscle fibers, whereas no distortion was observed in MA-NHS–linked tissues. Scale bars, 20 μm. acryloyl-X, SE, 6-((acryloyl)amino)hexanoic acid, succinimidyl ester; ExM, expansion microscopy; GA, glutaraldehyde; MA-NHS, methacrylic acid N-hydroxysuccinimide ester; μExM, expansion microscopy of microbes. (TIF) [file pbio.3000268.s004.tif]

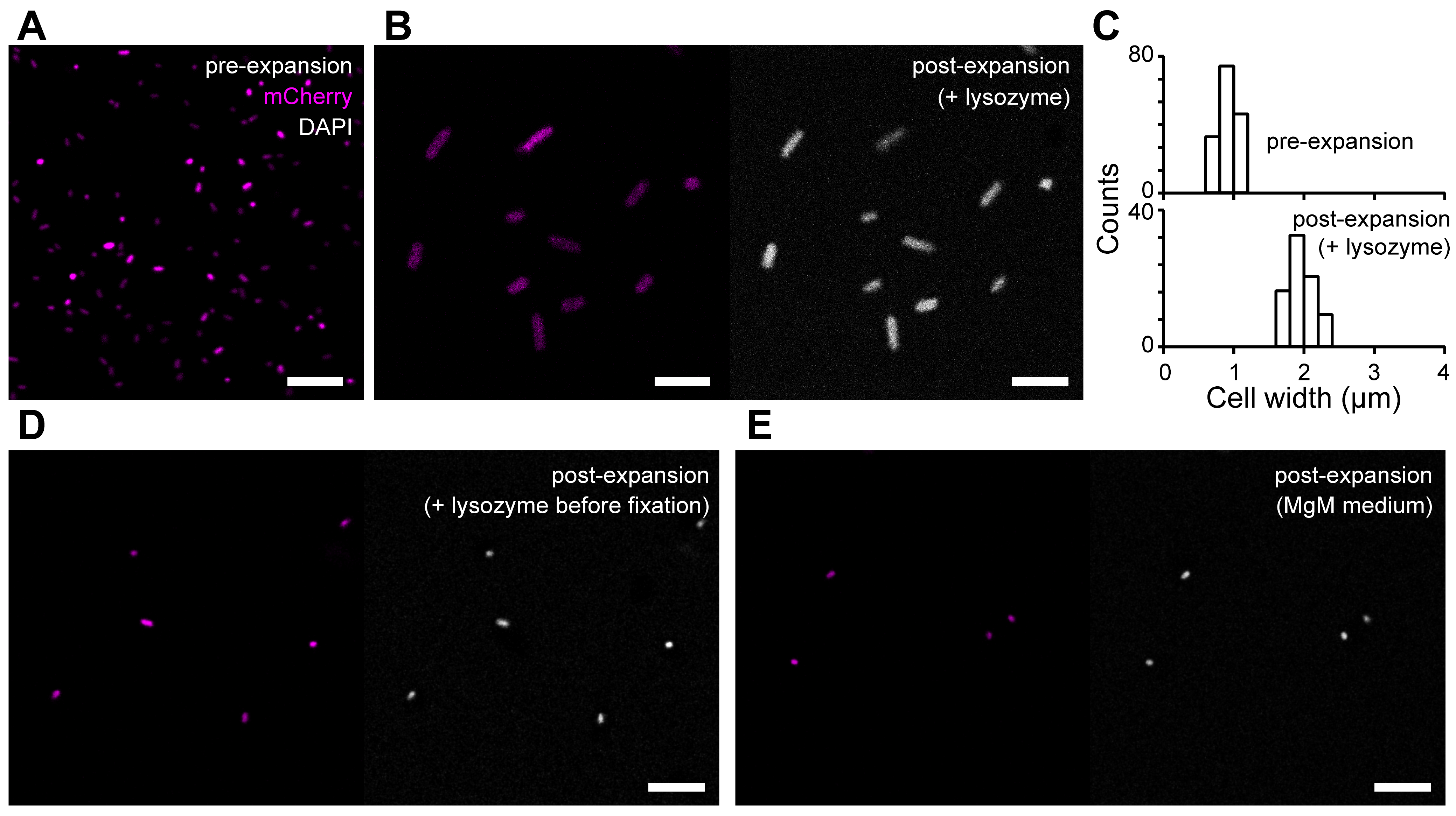

Supplement: S5 Fig — (A) Representative maximum intensity projection of mCherry-Salmonella cells before expansion. (B) After 1 h of lysozyme treatment to digest the cell wall, Salmonella cells expanded approximately 2-fold. Note that mCherry (left) and DAPI (right) signals colocalized. (C) Quantification of the expansion of cells in images similar to (B). The data underlying this figure are included in S11 Data. (D, E) Live cells that were treated with 0.5 mg mL−1 lysozyme for 1 h at 37°C prior to fixation (D) or cultured in an acidic, magnesium-depleted minimal medium (MgM-MES, pH 5.0, used to mimic the low pH, low Mg2+ environment of the phagosome) (E) did not expand, indicating that the cell wall remained intact under these conditions. Scale bars, 10 μm. MgM-MES, magnesium minimal MES medium; μExM, expansion microscopy of microbes. (TIF) [file pbio.3000268.s005.tif]
