# Supplementary material for: Mechanically resolved imaging of bacteria using expansion microscopy
Source: PLoS Biol. 2019 Oct 17;17(10):e3000268. doi: 10.1371/journal.pbio.3000268 (PMC6797083; doi:10.1371/journal.pbio.3000268)
Supplement: S1 Table — μExM, expansion microscopy of microbes. (DOCX) [file pbio.3000268.s006.docx]

**S1 Table: Reagents used in µExM.**

| Reagents | Vendor | Product # |
| --- | --- | --- |
| Ammonium persulfate (APS) | Thermo Scientific | 17874 |
| Sodium acrylate | Sigma-Aldrich | 408220 |
| 40% acrylamide solution | Bio-Rad | 161-0140 |
| 2% N,N'-methylenebisacrylamide solution | Bio-Rad | 161-0142 |
| Formaldehyde | Thermo Scientific | 28906 |
| Nonidet P 40 substitute (NP-40) | Sigma-Aldrich | 74385 |
| Triton X-100 | Fisher Scientific | BP151 |
| 4-hyroxy-TEMPO | Sigma-Aldrich | 176141 |
| Tetramethylethylenediamine (TEMED) | Thermo Scientific | 17919 |
| PBS, 20× | TEKNOVA | P0191 |
| Guanidine hydrochloride | Thermo Scientific | 24110 |
| Vancomycin | Sigma-Aldrich | V0045000 |
| Mutanolysin from *Streptomyces globisporus* | Sigma-Aldrich | M9901 |
| Lysozyme solution | Thermo Scientific | 90082 |
| Methacrylic acid N-hydroxysuccinimide ester | Sigma-Aldrich | 730300 |
| Proteinase K | NEB | P8107 |
| TO-PRO-3 iodide solution | Invitrogen | T3605 |
